# G-quadruplex ligand-mediated CHK1 and ERα suppression in endocrine-sensitive and endocrine-resistant luminal breast cancer cells

**DOI:** 10.1080/15384101.2026.2753856

**Published:** 2026-10-30

**Authors:** Martina Valenzuela, Valentina Pirota, Filippo Doria, Claudia Bellucci, Filippo Acconcia, Federico Dinoi, Erica Salvati, Francesco Berardinelli, Antonio Antoccia

**Affiliations:** a Department of Science, Roma Tre University, Rome, Italy; b Dipartimento di Chimica, Università di Pavia, Pavia, Italy; c Department of Biology and Biotechnology “C.Darwin”, Sapienza University of Rome, Rome, Itlay; d Institute of Molecular Biology and Pathology, National Research Council, Rome, Italy

**Keywords:** G-quadruplex, replication stress, breast cancer, G4-ligands, CHK1, ERα

## Abstract

G-quadruplex (G4)-stabilizing ligands are promising tools for inhibiting tumor cell growth in both in vitro and in vivo models. G4 stabilization interferes with DNA replication, leading to replication stress (RS) and DNA damage, and affects gene expression. Clinical development of the telomere-targeting G4 ligand RHPS4 was discontinued due to cardiotoxicity, prompting the synthesis of congeners with retained activity and improved toxicological profiles. In this study, the antiproliferative effects of RHPS4 derivatives were tested in breast cancer cell lines, MCF7, MDA-MB-231, and HCC1937, and compared with non-tumorigenic breast epithelial cells and normal fibroblasts. Among the analogues, compound 190 preferentially inhibited proliferation in MCF7 cells, an effect weakly associated with telomeric DNA damage. Similar to RHPS4, compound 190 induced RS, downregulated CHK1, and reduced *ESR1* mRNA and ERα protein levels in a dose-dependent manner. Reduced transcript levels and enhanced ERα degradation support contributions from both transcriptional and post-translational mechanisms, with degradation potentially linked to RS. These effects were also confirmed in endocrine-resistant MCF7-Y537S cells. Together, these findings identify CHK1 and ERα suppression as responses to compound 190 and support further investigation of acridine-based G4 ligands in endocrine-sensitive and endocrine-resistant luminal breast cancer.

## Introduction

It is well known that guanine-rich sequences may adopt secondary non-canonical structures known as G-quadruplexes (G4) which occur in both DNA [1] and RNA (mRNA as well as lncRNA) [2]. G4 structures may form spontaneously under physiological conditions [1,3,4], are thermodynamically stable, and exhibit extensive structural polymorphism [5,6]. One of the first biologically relevant G4-forming sequences studied was the telomere, composed of tandem G-rich repeats (5’-TTAGGG-3’ in vertebrates) [7]. Mammalian telomeres form a protective T-loop, stabilized by the shelterin complex [8], which maintains genomic stability, prevents chromosome degradation, and inhibits DNA repair processes [9]. In the last two decades, small ligands generated against telomeric G4 have emerged as a potential tool to fight cancerous cells, for their capability to inhibit telomerase and to dysfunctionalize telomeres [10–13].

Telomeric G4 ligands were proven to cause replication fork stall/collapse, DNA damage, cell cycle arrest, and *in vitro* impairment of cell growth of highly replicating tumor cells and cancer stem cells [14–18], whereas G4 ligands were less effective in normal primary cells [19–23]. This evidence was corroborated by *in vivo* studies performed on several cancer models demonstrating tumor control and remission over time [14,24–26].

Among G4 ligands, the pentacyclic acridine RHPS4 has proven particularly effective in blocking replication forks at telomeres, thereby inducing DNA damage, impaired cell proliferation, and cell death [16,27–29].

It is also known that RHPS4 displays off-target effects due to its ability to bind G4 located outside telomeres and in particular in gene promoters (*e.g. KDR*, which encodes for VEGFR-2) and gene bodies (*e.g. PROM1*, which encodes for CD133) [30,31]. Previous studies on glioma and osteosarcoma cells showed that RHPS4 is able to suppress the expression of CHK1, the central kinase of the replication stress response (RSR), governed by ATR [12,16].

Interestingly, overexpression of *CHEK1* is common in breast cancer (BC) as well as in many other cancers, including cervical, prostate, non-small cell lung, ovarian and pancreatic cancers, melanoma, and glioblastoma [32,33]. Overexpression is generally due to overactivation of the promoter in cancer cells [34,35] and is associated with poor prognosis and radio- and chemoresistance [33,36–39].

A possible explanation is that cancer cells overexpressing *CHEK1* are positively selected during tumor progression because of a survival advantage due to the increased ability to respond to replication stress (RS) induced by oncogene activation [40] and to repair DNA lesions induced by therapeutic agents [41,42]. It is worth noting that CHK1 is a potential therapeutic target in cancer including BC [43–46]. CHK1 inhibitors, including prexasertib and MK-8776, have been evaluated in preclinical studies of breast cancer [33,40,43] and have entered phase I/II clinical trials in patients with various malignancies (e.g. NCT05548296 for prexasertib and NCT01870596 for MK-8776).

Furthermore, G4-stabilizing compounds are toxic to homologous recombination (HR)-deficient cells (such as BRCA1-, BRCA2-, and RAD51-deficient cells) including those that develop resistance to PARP inhibitors (PARPi) [47,48]. Intriguingly, recent evidence suggested that CHK1 inhibitors affect estrogen receptor α (ERα) stability and content in luminal A and luminal B BC cells [40].

Luminal A and luminal B BCs typically express ERα, and patients with such tumors are generally treated with endocrine therapy (ET) drugs, which target ERα signaling. Classically, aromatase inhibitors (AI) (e.g. anastrozole), selective ERα modulators (SERMs like 4OH-tamoxifen) and selective ERα downmodulators (SERDs like fulvestrant) are used to treat ERα-positive BC patients.

Unfortunately, in approximately 20–30% of cases, prolonged patient treatment with ET drugs like AI or 4OH-tamoxifen determines the evolution of cell resistance to these drugs. Among the plethora of molecular mechanisms that sustain ET resistance, one mechanism of acquired ET resistance involves the selection of activating *ESR1* mutations, such as Y537S, which drive treatment resistance and disease recurrence [49]. Research is active to identify novel drugs that could target these mutated forms of ERα for alternative treatments of metastatic BC patients.

Although RHPS4 showed a promising preclinical profile, it was never tested in clinical trials due to cardiac toxicity observed in guinea pigs [50]. However, new derivatives of RHPS4 with reduced off-target effects and improved safety profiles have since been developed, characterized, and tested in colorectal cancer cells and SV40-immortalized fibroblasts [51]. From a chemical and structural standpoint, these analogues preserve the same RHPS4 aromatic rings but exhibit a variety of substituents at the 2- and 3-positions of the pentacyclic acridinium core, a modification able to modulate their activity [51] (Chart 1). In this paper, three RHPS4 congeners, herein named 190, 761, and 785 (previously named compounds 2, 6, and 8, respectively [51]), were tested for their capability to inhibit cell proliferation in a panel of cells representing different BC subtypes.

**Chart 1.**
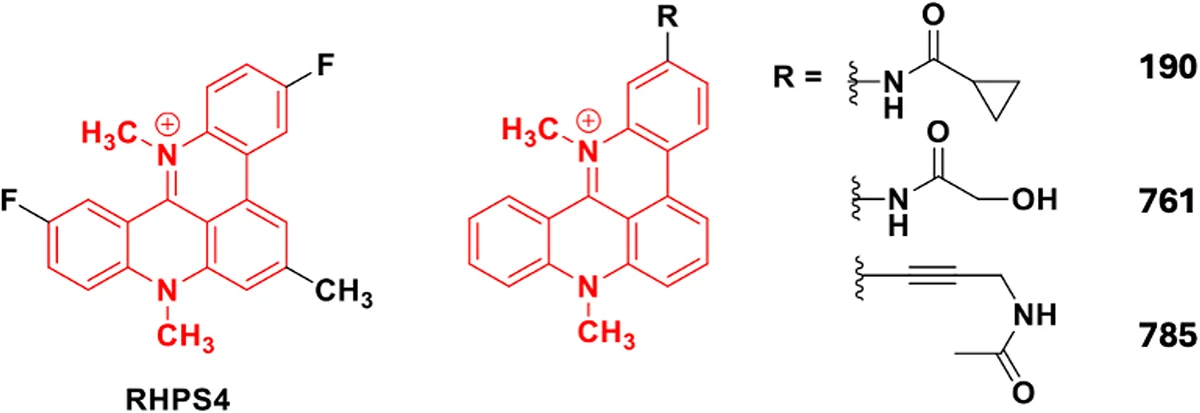
Structures of RHPS4 and related derivatives (190, 761, 785).

Among them, the most promising compound 190 was chosen for a deeper biological characterization.

Here we report that, although 190 exhibited a lower capacity to induce DNA damage (at both genomic and telomeric sites) than RHPS4, it showed the ability to induce RS, downregulate CHK1 expression, and reduce ERα protein levels in MCF7 cells and in a cell line modelling metastatic BC resistant to ET drugs (i.e. CRISPR/CAS9-engineered MCF7 cells expressing the ERα Y537S variant [52]).

## Results

### Cytotoxic effect of RHPS4 and its analogs in BC cell lines

In the first step of the study, we evaluated the cytotoxic efficacy of three different RHPS4 derivatives, namely 190, 761, and 785, in BC (MCF7, HCC1937, MDA-MB-231) and normal cell lines (MCF10A and MRC5). Exposure to increasing concentrations (ranging from 0.1 to 8 μM) of the compounds resulted in a remarkable and concentration-dependent inhibition of cell growth in almost all cancer cell lines. In particular, MCF7 cells showed the highest sensitivity to the ligands with IC_50_ values in the range between 0.25 and 4 μM (Figure 1(A–D)). In addition, among the BC cell lines analyzed, we observed the highest resistance in TNBC lines with IC_50_ values comparable to those of both normal cell lines (Figure 1(A–D)). All the RHPS4 derivatives have interesting properties but, in the present work, we decided to focus our attention on compound 190 due to its higher efficacy (lower IC_50_) in all the analyzed cell lines (Figure 1(E)).

**Figure 1.**
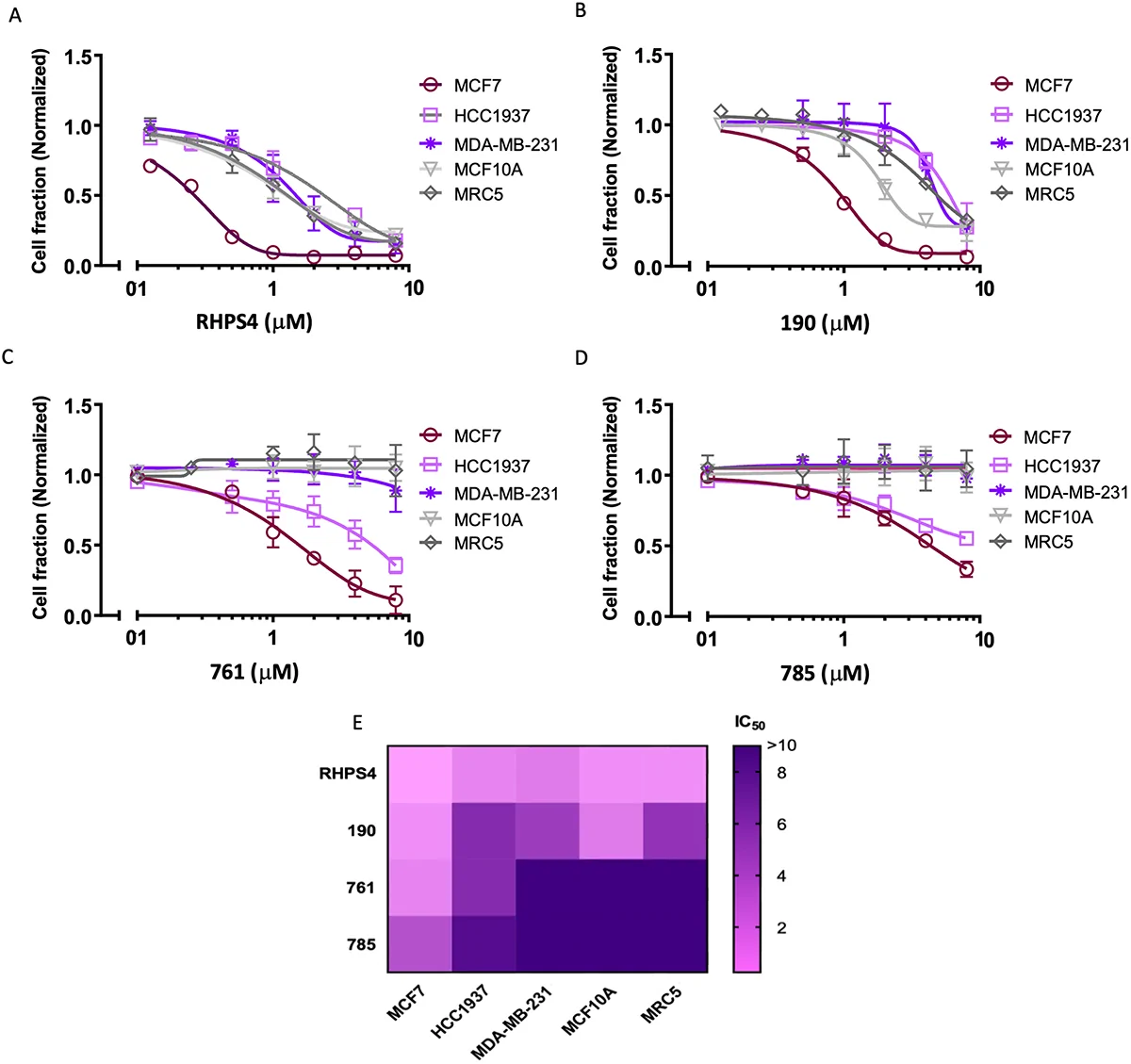
Cell proliferation inhibition following increasing concentrations of RHPS4 (A), 190 (B), 761 (C), and 785 (D) as assessed in BC (MCF7, HCC1937, MDA-MB-231) and normal cells (MCF10A and MRC5) (SRB data have been reported as cell fraction with respect to untreated cells and represent mean values ± SD, *N* = 3). (E) Heatmap showing RHPS4, 190, 761, and 785 IC_50_ as evaluated in a panel of BC and normal cell lines. The IC_50_ of each cell lines were represented using a color code from light pink to dark purple, covering the range 0–10 μM.

### Telomere length, DNA damage and telomere dysfunction in BC cell lines treated with compounds 190 and RHPS4

Resistance of cancer cell lines to G4 ligands has been associated with telomere length and telomere status [23,53]. Primarily, we analyzed basal telomere length in all BC cell lines using centromere-calibrated quantitative fluorescence in situ hybridization (Q-FISH) (Figure 2(A–D)). The mean basal values of telomere length were 15.2, 16.1, 36.5, and 17.5 T/C% in MCF7, MDA-MB-231, HCC1937, and MCF10A, respectively (Figure 2(E)). In addition, to evaluate the frequency of short telomeres in each cell line, distributions of telomere lengths were clustered in ten different groups (0–10; 11–20; 21–30; 31–40; 41–50; 51–60; 61–70; 71–80; 81–90; 91–100 T/C%) (Figure 2(F)). To assess if antiproliferative effects were associated with the induction of DNA damage and telomere dysfunction, γH2AX and TRF1 co-immunostaining was performed in BC and normal cells (Figure 2(G)). Our data confirm that RHPS4 is able to induce significant DNA damage in almost all BC cell lines both in genomic and telomeric regions, as previously reported for other cancer cell types [12,16,27–29,54]. Specifically, RHPS4 treatment caused a 2.6-, 1.4-, and 1.6-fold increase in genomic damage, while telomeric damage exhibited a 3.8-, 1.95-, and 1.3-fold increase in MCF7, HCC1937, and MDA-MB-231 cells, respectively (Figure 2(H,I)). Interestingly, RHPS4 did not induce DNA damage (neither genomic nor telomeric) in MCF10A epithelial control cells. On the contrary, 190 failed to induce DNA damage (with the exception observed in HCC1937 cells for genomic damage and in MCF7 cells for telomeric damage), displaying poor genotoxic properties in the analyzed cell lines (Figure 2(H,I)). The data collected showed a weak correlation between IC_50_ and either the mean telomere length or the frequency of short telomeres. However, it is interesting to note that the frequency of basal γH2AX foci and TIFs positively correlated with resistance to G4-ligands (Fig. S1).

**Figure 2.**
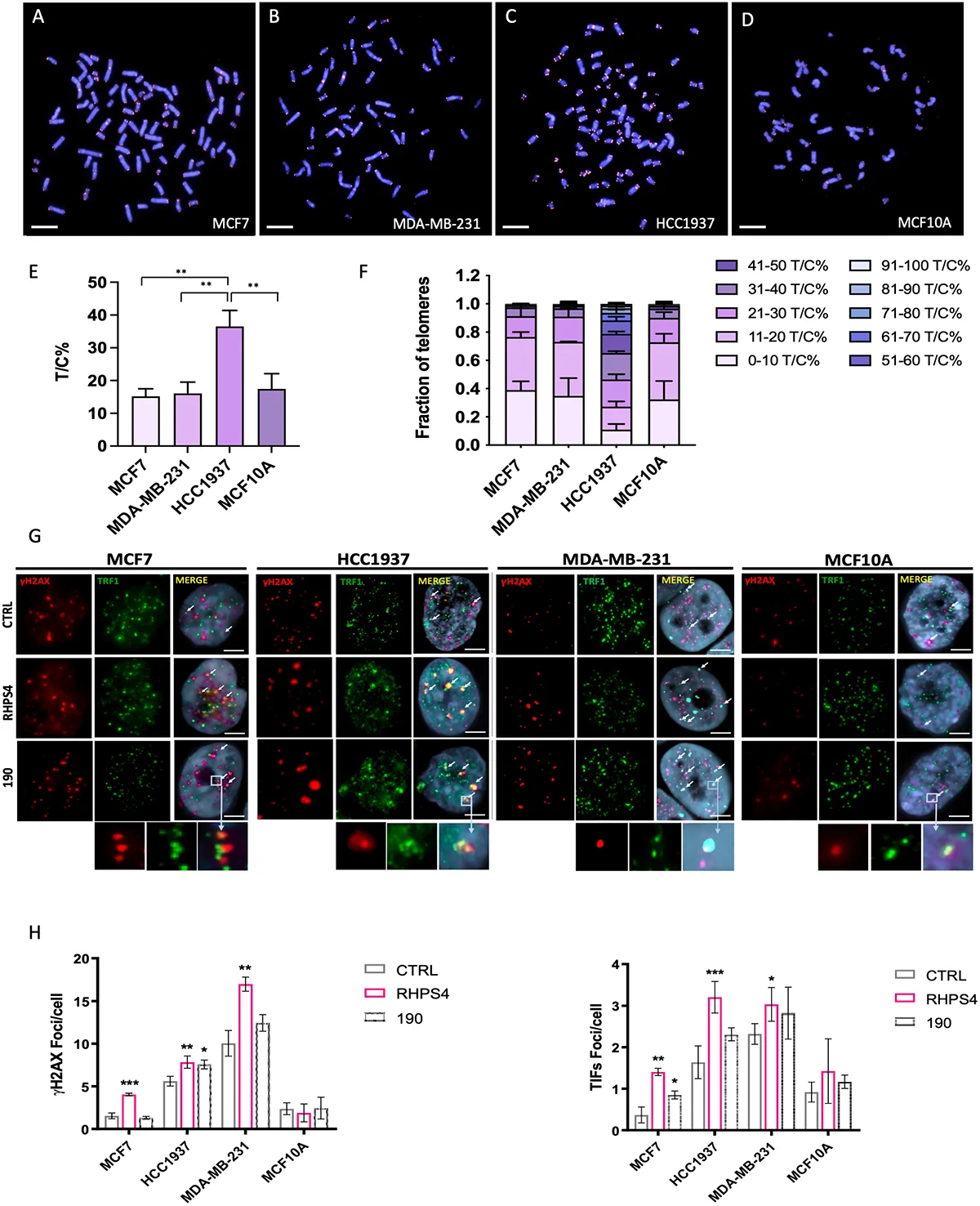
Representative images of MCF7 (A), MDA-MB-231 (B), HCC1937 (C), and MCF10A (D) metaphase spreads stained with telomeric and centromere 2Cy3-linked PNA probes and counterstained with DAPI (magnification 63×) (scale bar: 10 μm). (E) Mean telomere length in analyzed cell lines expressed as telomere fluorescence/centromere fluorescence × 100 (T/C%). (F) Histogram with stacked fraction of telomere lengths. Telomeres length was grouped in 10 different classes (0–10, 11–20, 21–30, 31–40, 41–50, 51–60, 61–70, 71–80, 81–90, 91–100 T/C%). Data have been reported as fraction of the total number of telomeres analyzed. Error bars represent SD (*N* = 2). Representative images of telomere dysfunction‑induced foci (TIFs) stained by immunofluorescence in BC cells lines MCF7, HCC1937, MDA-MB-231, and normal MCF10A cells (G) treated with RHPS4 or 190 at their respective IC_50_. For each cell line and condition, single channels (γH2AX signals in red and TRF1 telomeric protein signals in green) and merged images are illustrated. Arrows indicate γH2AX and TRF1 colocalizations (scale bar: 5 μm). Quantification of γH2AX foci (H) and TIFs (I) in BC and normal cells. Data have been reported as number of foci per cell and represent mean values ± SD (*N* = 3). *p < 0.05, **p < 0.01, ***p < 0.001 Student’s t-test.

### RHPS4 and 190 treatments decrease the levels of CHK1 protein in MCF7 cells

As previously reported for glioma cells [16], RHPS4 significantly reduced CHK1 protein levels in BC cells as well (Figure 3(A)). Specifically, we observed a 40% downregulation of CHK1 in MCF7 cells (Figure 3(B)) and a 60% downregulation in HCC1937 cells (Fig. S2). Similarly, compound 190 showed a comparable effect, downregulating CHK1 levels in both cell lines, with protein level reductions of 48% and 38% in MCF7 and HCC1937 cells, respectively (Figure 3(B) and Figure S2).

**Figure 3.**
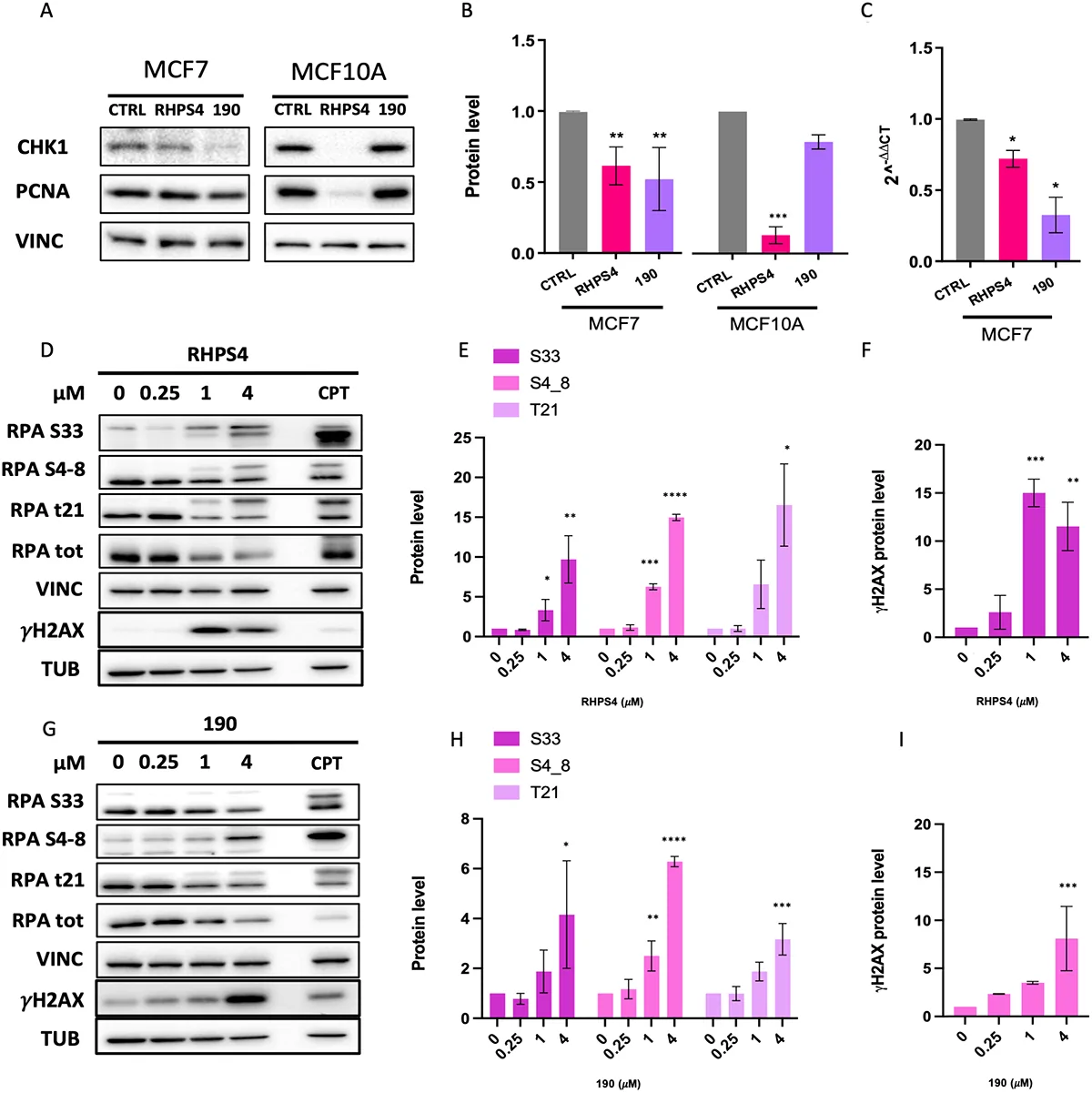
(A) Representative western blot showing CHK1 and PCNA protein amounts in untreated (CTRL) and G4 ligands-treated cells. Vinculin (VINC) was used to ensure for equal protein loading. Quantification of CHK1 (B) protein amounts after 190 and RHPS4 treatment. Data have been reported as relative protein levels with respect to untreated cells and represent mean values ± SD (*n* = 3). Quantification of *CHEK1* (C) mRNA expression in response to either 190 or RHPS4. Data have been reported as relative quantity with respect to untreated cells, according to the 2^−ΔΔCt^ method, and represent mean values ± SD (*n* = 3). Representative western blot showing total RPA, RPA S33, S4-8 T21 phosphorylation and γH2AX protein amounts in untreated (CTRL) and RHPS4 (D) and 190 (G)‑treated cells. Vinculin (VINC) and tubulin (tub) were used to ensure for equal protein loading. Quantification of RPA S33, S4-8 T21 phosphorylation (E,H) and γH2AX (F,I) protein amounts after RHPS4 and 190 treatments. Data have been reported as relative protein levels with respect to untreated cells and represent mean values ± SD (*N* = 3).*p < 0.05, **p < 0.01, ***p < 0.001, ****p < 0.0001 (one‑way ANOVA test and Tukey’s multiple comparison test).

The S-phase-specific protein PCNA was used as a control to ensure that CHK1 reduction was not caused by G4 ligand-induced S-phase depletion. This was further validated through cytofluorimetric analysis of BrdU-labeled BC cancer cells, which revealed only a slight reduction in S-phase cells and an overall delay in cell cycle progression (Fig. S3).

In the control cell line MCF10A, 190 failed to reduce CHK1 levels, whereas RHPS4 markedly reduced CHK1 protein levels (Figure 3(B)). However, it is worth noting that PCNA was also strongly depleted (Figure 3(A)), suggesting that the observed CHK1 depletion was dependent on S-phase abrogation. Interestingly, in BC cell lines the reduction in CHK1 protein levels was accompanied by decreased *CHEK1* mRNA expression (Figure 3(C) and Figure S2).

### Both RHPS4 and 190 determine a replication stress response in MCF7 cells

One of the well-known effects of RHPS4 treatment is the activation of RS and the DNA damage response, as demonstrated in several cell lines [27,28]. To determine whether compound 190 could induce a similar response in MCF7 cells, the phosphorylation status of the proteins RPA and H2AX was assessed. The data revealed that treatment with 1 µM and 4 µM RHPS4 strongly activated RPA phosphorylation at serine 33, serine 4–8, and threonine 21 (Figure 3(D,E)).

In contrast, compound 190 induced a lower activation of RPA phosphorylation, which significantly increased at 1 µM (S4_8) and 4 µM (S33, S4_8, and T21) (Figure 3(G,H)). Western blotting (WB) analyses of γH2AX confirmed this trend, showing a stronger effect upon RHPS4 treatment, consistent with findings obtained from our immunofluorescence analysis (Figure 3(F,I)).

### *CHEK1* is overexpressed in BC and correlates with relapse free survival (RFS) in BC patients

Figure 4 shows the mRNA levels of *CHEK1* in 22 different types of human cancer, based on TCGA data. The data indicate a consistent trend in gene expression, with higher levels of *CHEK1* mRNA observed in various types of cancer compared to normal tissues (Figure 4(A)). When we analyzed data from the database for BC patients, the results clearly showed the mRNA level of *CHEK1* was higher in BC tissues (1097 cases) than in non-BC tissues (493 cases) (Figure 4(B)).

**Figure 4.**
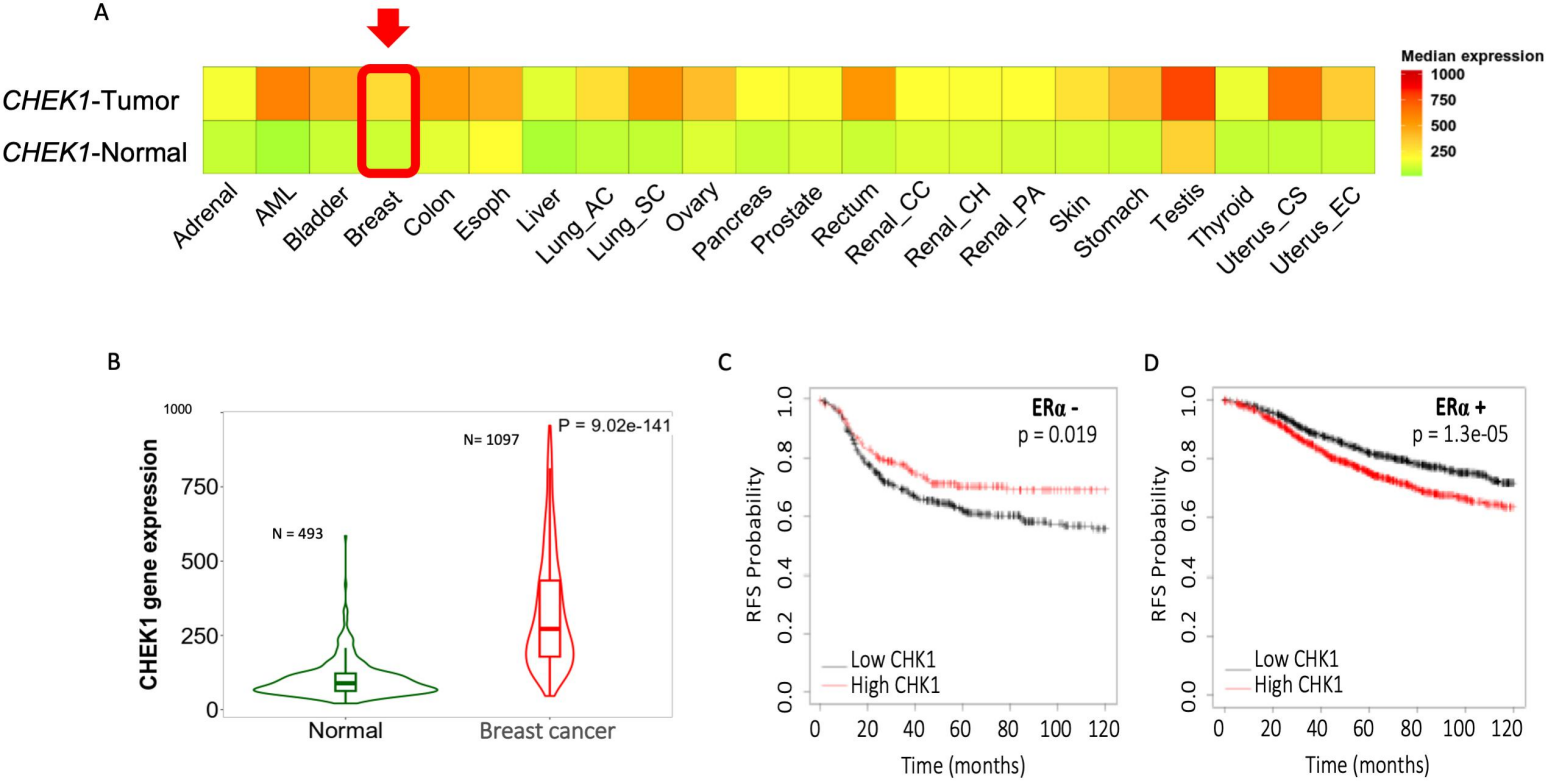
(A) Heatmap representing the mRNA levels of *CHEK1* in 22 types of tumor tissues and non-cancer tissues retrieved by the https://tnmplot.com/analysis (accessed on 01/02/2025) web portal. The mRNA expression levels of *CHEK1* (B) in both BC and normal individuals, based on TNM. Comparison between the relapse free survival (RFS) probability curve of breast cancer patients overexpressing or not *CHEK1* in relation to ERα status (ERα- (C) and ERα+ (D)). The red line represents BC patients harboring tumors with high *CHEK1* expression, and the black line represents BC patients with low *CHEK1* expression.

Regarding prognostic value, *CHEK1* mRNA levels showed a significant association with relapse free survival (RFS) in ERα-positive compared to ERα-negative BC patients (Figure 4(C,D)). Specifically, high *CHEK1* levels negatively correlates with RFS in ERα-positive BC patients, while ERα-negative patients exhibited an opposite trend.

### RHPS4 and 190 induce ERα depletion in MCF7 cells

Previous work has demonstrated that in BC cells, ERα is depleted following the induction of replication stress [40]. Because RHPS4 and 190 induced the activation of RS, we next evaluated their effect on receptor intracellular levels in MCF7 cells. As a control, MCF7 cells were additionally treated with camptothecin (CPT), a well-known inducer of RS [55]. Both ligands were able to induce a dose-dependent reduction of the ERα although with a different efficiency. A significant reduction upon RHPS4 treatment was observed at concentrations as low as 0.25 µM, whereas 1 µM was necessary to observe a significant reduction following 190 treatments of MCF7 cells (IC_50_ values were about 0.4 and 1.2 µM for RHPS4 and 190, respectively) (Figure 5(A,B)). Interestingly, these concentrations fit very well with respective IC_50_ values observed in MCF7 cells, supporting the correlation of antiproliferative effects and ERα reduction (Fig. S4). Moreover, this reduction was accompanied by RPA and γH2AX phosphorylation, suggesting that these events may be interconnected (Fig. S4). A similar reduction in ERα levels was observed following treatment with the CHK1 inhibitor prexasertib (Figure 5(C,D)), consistent with previous reports that CHK1 inhibition promotes ERα degradation [40]. To determine whether increased protein degradation contributes to the reduction in ERα levels following G4 ligand treatment, we investigated ERα protein turnover. MCF7 cells were pre-treated with the protein synthesis inhibitor cycloheximide (CHX) for 2 h before the administration of RHPS4 and 190 for 24 h. Interestingly, both ligands are able to determine a significant CHX-dependent reduction in ERα intracellular levels, suggesting that G4 ligands also regulate ERα abundance at the post-translational level (Fig. S5). However, it is also well known that G4 ligands can also determine gene expression downregulation through the direct binding of G4 sequences in the gene promoter. To determine whether the regulation responsible for the reduction in ERα levels occurred at the transcriptional level, RT–PCR experiments were performed (Figure 5(E,F)). The results showed that both ligands were able to significantly downregulate *ESR1* gene expression at concentrations of 1 and 4 µM, for RHPS4 and 190, respectively. This evidence suggests that G4-dependent *CHEK1* downregulation is accompanied by a significant reduction in ERα protein levels, resulting from both transcriptional and post-transaltional regulation.

**Figure 5.**
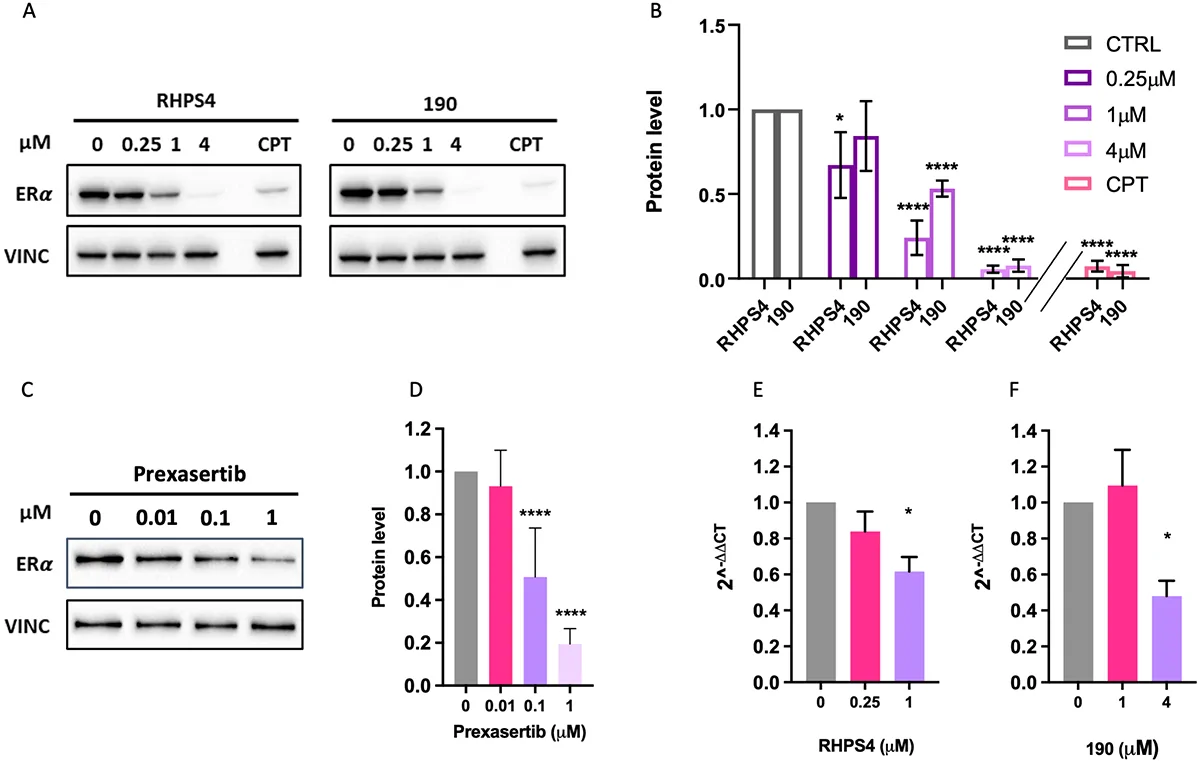
Representative western blot showing ERα protein amounts in untreated, G4 ligands-treated MCF7 cells. Campthotecin (CPT) was used as validated replication stress inducer (A). Vinculin (VINC) was used to verify the equal protein loading. Quantification of ERα (B) protein amounts after RHPS4, 190 and camptothecin (CPT) treatment in MCF7 cells. Data have been reported as relative protein levels with respect to untreated cells and represent mean values ± SD (*N* = 3). Representative western blot showing ERα protein amounts in untreated and prexasertib-treated MCF7 cells (C). Quantification of ERα (D) protein amounts after prexasertib treatment in MCF7 cells. Data have been reported as relative protein levels with respect to untreated cells and represent mean values ± SD (*N* = 6). mRNA expression of the *ESR1* gene in response to either RHPS4 (E) or 190 (F). Data have been reported as relative quantity with respect to untreated cells, according to the 2^−ΔΔCt^method, and represent mean values ± SD (*N* = 3). *p < 0.05, **p < 0.01, ***p < 0.001, ****p < 0.0001 (Student’s*t*-test).

### Both RHPS4 and 190 reduce CHK1 and ERα levels in a cell line modelling metastatic BC resistant to ET drugs

To evaluate the sensitivity of ET-resistant cells to G4 ligand treatment, MCF7-Y537S cells were treated with RHPS4 and 190, followed by analysis of CHK1 and ERα protein and RNA levels. The data demonstrated that MCF7-Y537S cells exhibited comparable sensitivity to both RHPS4 and 190, with an IC_50_ of approximately 1 µM (Figure 6(A,B)). Interestingly, MCF7-Y537S cells showed greater sensitivity to treatment (particularly with 190) compared to TNBC cells and control cells (Figure 6(A,B)). Furthermore, both RHPS4 and 190 significantly reduced CHK1 protein and mRNA levels without affecting PCNA levels or depleting S-phase (Figure 6(C–E) and Figure S3). Finally, both ligands mediated a dose-dependent reduction of ERα levels, as assessed at both the protein (IC_50_ values were about 0.3 and 1.6 µM for RHPS4 and 190, respectively) (Figure 6(F,G)) and mRNA levels (Figure 6(H,I)) in ET-resistant cells. As outlined above, CPT and prexasertib were used as controls for the induction of replication stress and CHK1 inhibition (Figure 6(F,G,J,K)).

**Figure 6.**
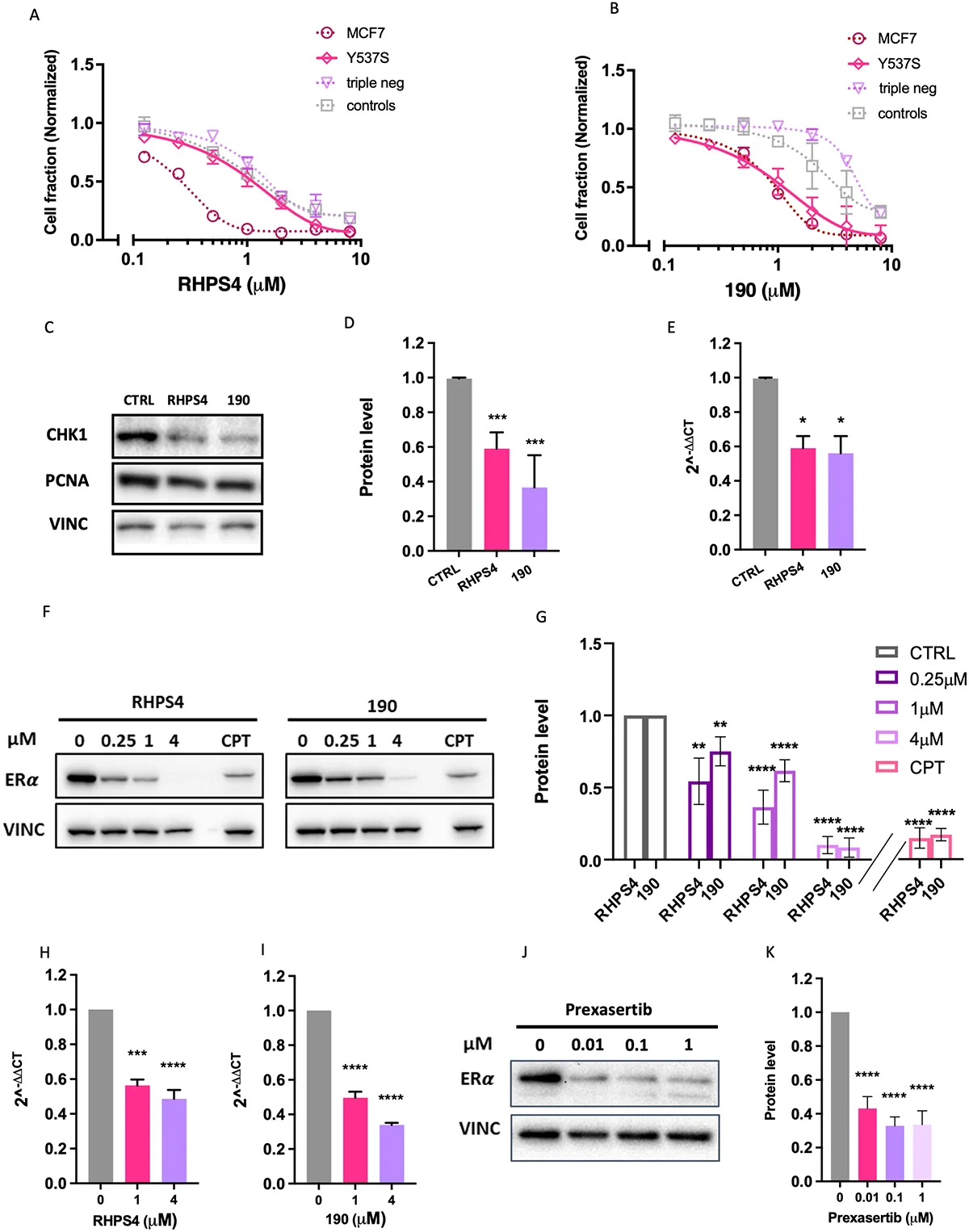
Cell proliferation inhibition following increasing concentrations of RHPS4 (A) and 190 (B) as assessed in MCF7-Y537S (and compared with MCF7, triple-negative BC and normal cells). Data have been reported as cell fraction normalized on untreated cells and represent mean values ± SD, *N* = 3. Representative western blot showing CHK1 and PCNA protein amounts in untreated (CTRL) and G4 ligands-treated MCF7-Y537S cells (C). Vinculin (VINC) was used to ensure for equal protein loading. Quantification of CHK1 (D) protein amounts after 190 and RHPS4 treatment. Data have been reported as relative protein levels with respect to untreated cells and represent mean values ± SD (*N* = 3). Quantification of *CHEK1* mRNA expression upon RHPS4 or 190 exposures (E). Data have been reported as relative quantity with respect to untreated cells, according to the 2-ΔΔCt method, and represent mean values ± SD (*N* = 3). Representative western blot showing ERα protein amounts in untreated, G4 ligands-treated Y537S cells. Campthotecin (CPT) was used as validated replication stress inducer (F). Vinculin (VINC) was used to verify the equal protein loading. Quantification of ERα (G) protein amounts after RHPS4, 190 and CPT treatment in Y537S cells. Data have been reported as relative protein levels with respect to untreated cells and represent mean values ± SD (*N* = 3). mRNA expression of the *ESR1* gene in response to either RHPS4 (H) or 190 (I) in Y537S cells. Data have been reported as relative quantity with respect to untreated cells, according to the 2^−ΔΔCt^ method, and represent mean values ± SD (*n* = 3). Representative western blot showing ERα protein amounts in untreated and prexasertib-treated Y537S cells (J). Quantification of ERα protein amounts after prexasertib treatment in Y537S cells (K). Data have been reported as relative protein levels with respect to untreated cells and represent mean values ± SD (*N* = 6). *p < 0.05, **p < 0.01, ***p < 0.001, ****p < 0.0001 (Student’s *t*-test).

### Promoters of *CHEK1* and *ESR1* harbor sequences able to fold into G4 structures

The principal mechanism responsible for G4-mediated gene expression downregulation is the binding of G4 ligands to G4 structures in the promoter region of such genes. To confirm the ability of the ligands to bind G4 structures present in the *ESR1* and *CHEK1* promoters, the sequences *CHEK1* (5′-GGGACGGGTGGGGCTGTGGATGGGA-3′) and *ESR1* (5′-GGGAGGGAGGGAGGGAGGGAGAAGGG-3′), identified by bioinformatic analysis as putative G4-forming sequences (Figure 7(A,B)), were characterized by circular dichroism (CD) spectroscopy under various ionic conditions (10, 20, and 100 mM KCl) in 10 mM lithium cacodylate buffer (pH 7.4), while maintaining a constant ionic strength of 100 mM through the addition of LiCl (Figure 7(C,D)). Specifically, the CD spectra of *CHEK1* exhibited a main positive band at 264 nm accompanied by a shoulder around 290 nm and a negative band at 240 nm, suggesting a hybrid G4 topology (Figure 7(C)), while *ESR1* displayed a CD profile consistent with a parallel G4 topology, with a strong positive maximum around 264 nm and a negative minimum near 240 nm (Figure 7(D)). For *CHEK1*, the ellipticity intensity increased with potassium concentration, suggesting enhanced stabilization of the G4 fold at higher K^+^ levels. *ESR1* displayed similar spectral features, although with higher overall ellipticity values, consistent with a more stable or compact G4 arrangement (Figure 7(C,D)).

**Figure 7.**
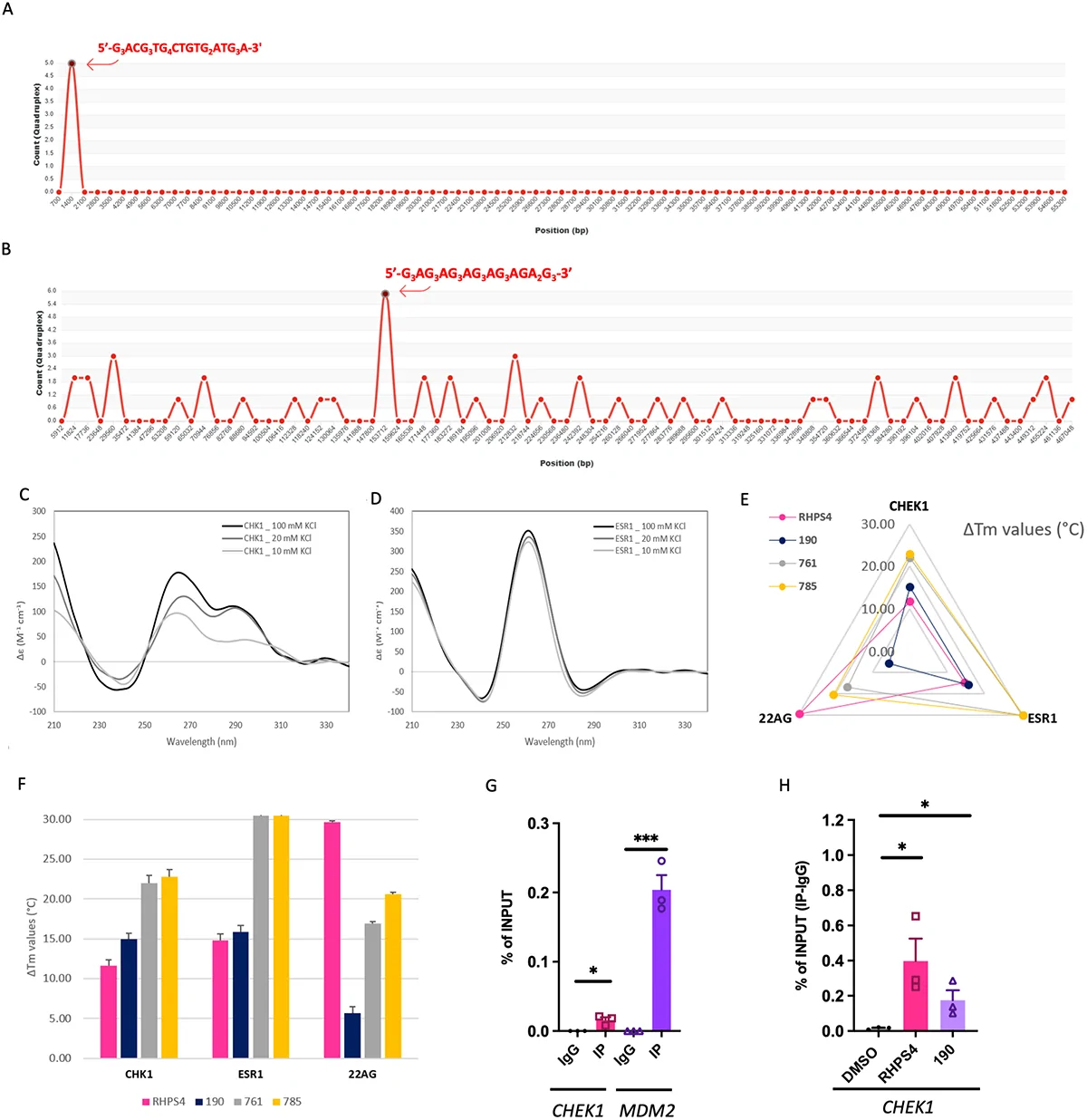
(A) Predicted G-quadruplex sequences (PQS) identified by G4Hunter (window size: 25; threshold: 2.0) within the nucleotide region Chr11: 125624800-125681124 of *Homo sapiens*. The *CHEK1* promoter core region (Chr11:125624800-125628201) corresponds to positions 1–3402 in the present image. The PQS identified in that maximum with the highest G4Hunter score (=2.038) is indicated by the red arrow. (B) Predicted G-quadruplex sequences (PQS) identified by G4Hunter (window size: 25; threshold: 2.0) within the nucleotide region Chr6:151656672-152129619 of *Homo sapiens*. The *ESR1* promoter PQS with the highest G4Hunter score (=2.115) is indicated by the red arrow. CD spectra of 2.5 μM *CHEK1* (C) or *ESR1* (D) in 10 mM lithium cacodylate buffer, pH 7.4, with different 10, 20, or 100 mM KCl. The ionic strength was maintained constant at 100 mM by adding the proper amount of LiCl. (E and F) ∆tm values were obtained in the presence of 2.5 µM oligonucleotides (*CHEK1, ESR1*, and 22AG) with 4 equiv. of RHPS4, 190, 761, or 785 (10 µM) in 10 mM lithium cacodylate buffer, pH 7.4, with 10 mM KCl for *ESR1* and 20 mM kcl for *CHEK1* and 22AG. The ionic strength was maintained constant to 100 mM by adding the proper amount of LiCl. Data are presented as a radar plot (E) and histogram (F). Chromatin immunoprecipitation assay with BG4 antibody in untreated (G) or RHPS4 and 190 treated MCF7 (H). Immunoprecipitated samples were analyzed by real-time qPCR with specific primers against the G4-containing regions of *CHEK1* promoter or *MDM2* as a positive control. Data represent mean values ± SD (*N* = 3). *p < 0.05, ***p < 0.001 (Student’s *t*-test).

To further support the structural assignment, PCA analysis was performed by comparing the CD spectra of *ESR1* and *CHEK1* with those of G4s of known topologies (Fig. S6). The resulting score plot showed that *ESR1* clustered with parallel G4s, while *CHEK1* localized within the region associated with hybrid G4s, in close proximity to reference structures such as 2JSM and 2HY9.

Together, these results support G4 folding of the *CHEK1* and *ESR1* sequences under the tested *in vitro* conditions.

To evaluate the intrinsic thermal stability of the newly identified *ESR1* and *CHEK1* G4s, we performed both CD- and UV-melting experiments under controlled ionic conditions (Table 1, Figure S7 A–D, and S8). The melting temperatures (Tm) were recorded for each sequence in 10 mM lithium cacodylate buffer, pH 7.4, containing different concentrations of KCl, while maintaining the total ionic strength constant at 100 mM by addition of LiCl. Thermodynamic parameters (ΔH° and ΔS°) were derived from the UV-melting profiles within the linear range of the van’t Hoff plots (0.03 < θ <0.97). Both techniques revealed consistent melting temperatures, confirming the formation of stable G4 structures for the two sequences (Table 1). The thermodynamic analysis showed negative enthalpy and entropy changes (ΔH° ≈ −50 to −56 kcal mol^−1^; ΔS° ≈ −140 to −170 cal mol^−1^ K^−1^; Table 1), typical of G4 formation processes driven by favorable base-stacking and cation coordination balanced by loss of conformational freedom. The overall good agreement between CD- and UV-derived Tm values further validates the robustness of these measurements.

**Table 1.** Melting temperatures (Tm) determined by CD and UV melting experiments for 2.5 µM *ESR1* and *CHEK1* G4s in 10 mM lithium cacodylate buffer (pH 7.4) at varying potassium concentrations. The total ionic strength was kept constant at 100 mM by adding appropriate amounts of LiCl. Thermodynamic parameters (ΔH° and ΔS°) were obtained from UV-melting analyses.

		CD-melting	UV-melting		
	[KCl] (mM)	Tm (°C)	Tm (°C)	ΔH (Kcalmol^−1^)*	ΔS (calmol^−1^K^−1^)*
*ESR1*	5	49.0 ± 0.1	53.2 ± 0.2	−50 ± 2	−154 ± 5
	**10**	**64.5 ± 0.2**	**65.2 ± 0.1**	−53.9 ± 0.9	−160 ± 3
*CHEK1*	10	54.6 ± 0.4	55.8 ± 0.1	−56.1 ± 0.5	−170 ± 2
	**20**	**65.7 ± 0.3**	**61.9 ± 0.3**	−47 ± 2	−141 ± 5

*This analysis should be restricted to the temperature range for which 0.03<ø<0.97.

These data establish *ESR1* and *CHEK1* as thermodynamically stable, K^+^-dependent G4 structures, suitable for subsequent ligand-binding studies.

### RHPS4 and 190 bind G4 structures in *CHEK1* and *ESR1* promoters

To assess the ability of small molecules to stabilize the newly identified *ESR1* and *CHEK1* G4s, we performed CD-melting experiments in the presence of the four ligands: RHPS4, 190, 761, and 785. Based on these results, the ionic conditions yielding a melting temperature around 65°C (10 mM KCl for *ESR1* and 20 mM KCl for *CHEK1*) were selected for ligand-induced stabilization assays. Telomeric 22AG G4 (5’-AGGGTTAGGGTTAGGGTTAGGG-3’) was used as a reference, since it constitutes the predominant and best-characterized G4 structure in the human genome. Its melting temperature, determined by CD-melting experiments in 20 mM KCl, was 56.05 ± 0.07°C, in agreement with previously reported values (Fig. S7E) [56].

Ligands were added at 4 equiv. (10 µM) and incubated for 4 h at RT before recording CD spectra (Fig. S9) and melting.

The thermal stabilization data (Tables S1–S2) revealed distinct ligand-dependent stabilization profiles across the three G4 targets (Figure 7(E,F)). RHPS4, a known telomeric binder, strongly stabilized 22AG (ΔTm ≈ 30 °C) but showed only moderate effects on *ESR1* (ΔTm ≈ 15 °C) and *CHEK1* (ΔTm ≈ 12 °C), confirming its poor selectivity and preference for canonical telomeric G4s. In contrast, the RHPS4-derived compounds (190, 761, and 785) displayed improved selectivity for the gene-associated G4s. Both 761 and 785 induced substantial stabilization of *ESR1* (ΔTm up to 30 °C) and *CHEK1* (ΔTm ≈ 20 °C), while having a reduced effect on 22AG.

Among these, 190 emerged as the best-performing ligand, providing a balanced stabilization profile with good ΔTm values on *ESR1* and *CHEK1* (ΔTm ≈ 16 °C) but minimal impact on the telomeric control (ΔTm ≈ 6 °C). This selective stabilization pattern suggests that 190 retains the core G4-binding capability of RHPS4 while displaying a reduced affinity for off-target telomeric structures, an important step toward achieving topology- and sequence-selective G4 modulation. To assess the in-vivo capacity of CHK1 promoter to fold into a stable G4 structure in the chromatin environment, we performed chromatin immunoprecipitation with the anti-DNA and RNA G4 structures (BG4) specific antibody. As shown, we observed a significant enrichment in *CHEK1* promoter DNA sequences in BG4 immunoprecipitated samples, compared to IgG precipitated samples, as evidenced by real-time qPCR results (Figure 7(G)). The result is comparable to the enrichment observed in the same samples on MDM2 promoter (which is a well-known site for G4 structures, used as a positive control [57]). Moreover, coherently with their stabilizing effect, the treatment with both RHPS4 and 190 induced a further increase of enrichment (Figure 7(H)).

## Discussion

In the last decades, increasing interest has grown around telomeric targeting thanks to a larger availability of new telomere-targeting agents such as telomeric G4 ligands. Among them, RHPS4 was one of the most promising, effective, and characterized molecules [27,55,58–60] but *in vivo* preclinical studies revealed a dose-related decrease in heart rate and in QT interval corrected for heart rate at all doses of treatment [50]. For this reason, RHPS4’s path to clinical trials was halted, and new derivatives were synthesized, preserving the pentacyclic acridinium core but introducing different substituents at the 2- and 3-positions [51] with the aim of minimizing cardiotoxicity. In the present work, we investigated three previously developed RHPS4 analogues [51] to identify compounds with antiproliferative activity in BC cells and to explore the contribution of telomere dysfunction and modulation of *CHEK1* and ERα expression to their biological effects, including in a model of endocrine therapy resistance.

Data obtained in the present study showed a mean RHPS4 IC_50_ that is in good accordance with previously reported data for BC cells [24,58,61]. Specifically, RHPS4 exhibited antiproliferative effects even at low concentrations, as observed in ERα-positive MCF7 cells. In contrast, the triple-negative cell lines HCC1937 (BRCA1-mutated) and MDA-MB-231 showed greater resistance to the treatment. Among the RHPS4 analogues, 761 and 785 displayed the lowest effectiveness in inhibiting cell proliferation, whereas 190 demonstrated the highest efficacy. Consistent with the results obtained for RHPS4, triple-negative cell lines HCC1937 and MDA-MB-231 were significantly more resistant to 190 treatments compared to MCF7 cells.

The greater resistance of HCC1937 and MDA-MB-231 cells was accompanied by higher basal levels of γH2AX. Elevated pretreatment γH2AX levels have previously been associated with reduced chemotherapy response in TNBC and poorer survival in chemotherapy-treated BC patients [62,63]. Several factors may explain the higher levels of constitutive DNA damage observed in BRCA1-deficient and TNBC cells, including RS, mutations in DNA repair genes, and telomeric defects [64–66].

Consistent with this observation, both HCC1937 and MDA-MB-231 cells exhibited basal levels of γH2AX foci that were 3–4 times higher than those observed in MCF7 cells.

All the tested ligands (RHPS4, 190, 761, and 785) showed greater antiproliferative activity in MCF7 cells than in the non-tumorigenic control cells MCF10A and MRC5, consistent with previous findings reported for RHPS4 [27,29,60]. In contrast, the triple-negative BC cell lines generally showed sensitivity comparable to that of the control cells. Among the three RHPS4 derivatives, 190 consistently showed the lowest IC_50_ values across the BC cell lines tested and was, therefore, selected for further characterization of its mechanism of action. Although it was originally reported that 190 maintains the same pharmacological profile and the same mechanism of action as its precursor [51], our data suggest a milder efficacy in DNA damage induction in BC cells. Indeed, when comparing the genotoxic potential of RHPS4 and 190, we observed that the derivative compound was less effective in inducing both genomic and telomeric DNA damage. These findings raise questions about the mechanism through which 190 inhibits cell proliferation in BC cells and motivate further investigation into its mode of action, including an analysis of the transcriptional status of known RHPS4 target genes, such as *CHEK1*.

The ATR-CHK1-mediated DNA damage response is generally thought to be the main replication stress-responsive pathway that mediates activation of the DNA damage checkpoint in S-phase [67,68]. Interestingly, in recent years *CHEK1* has been found to be overexpressed in BC as well as many other cancer types.

Assessing the survival rates of cancer patients in relation to *CHEK1* expression levels indicates that high levels of expression in BC are associated with lower chances of survival [36,69,70]. When this gene is overexpressed in cancer cells, there is a noticeable increase in HR activity, which can confer resistance to conventional cancer treatments [35,43,69]. Therefore, inhibiting the expression of *CHEK1* in HR-proficient tumor cells could potentially enhance the effectiveness of current therapies, and could also help restore synthetic lethality in HR-deficient tumors that have become resistant to PARP inhibitors. Interestingly, *CHEK1* seems to be crucial in the response to DNA damage-inducing agents in BC and glioma cells [39,71]. In previous studies, we showed that RHPS4 treatment reduced CHK1 protein levels in glioma cells [16,29]. Here, we extend this observation to BC cells treated with either RHPS4 or 190, where reduced protein levels were accompanied by a decrease in *CHEK1* mRNA. Our biophysical analyses showed that a *CHEK1* promoter-derived sequence folds into a G4 structure that can be stabilized by both ligands in vitro. BG4 ChIP experiments further supported the presence of G4 structures within this promoter region in chromatin, including following ligand treatment. Together, these findings support a model in which ligand-mediated stabilization of the CHK1 promoter G4 contributes to the transcriptional downregulation of *CHEK1*. Although *CHEK1* downregulation has been implicated in the radiosensitization of glioma cells [72,73], surviving fraction experiments did not reveal any sensitization to X-rays in BC cell lines (Fig. S10). Thus, the reduction in CHK1 expression induced by these ligands did not translate into a detectable radiosensitizing effect in our models. Beyond its role in the radiation response, CHK1 has also been linked to hormone receptor status and endocrine resistance in BC. Previous studies reported an association between CHK1 expression and ER/PR status, as well as increased CHK1 expression in PDX-derived and experimentally engineered ET-resistant BC cell lines [69]. Moreover, treatment with CHK1 inhibitors promoted ERα degradation in ERα-expressing BC cells [40].

Interestingly, analysis of CHK1 expression in BC progression revealed an inverse correlation between its presence and relapse-free survival (RFS) probability in women with ERα-positive BC. Although the molecular mechanism linking CHK1 inhibition and ERα degradation has not yet been characterized, it seems to be related to RS as the induction of RS by different compounds (including CPT), but not the induction of DNA damage, was sufficient to induce a reduction in ERα intracellular levels in BC cells [40].

These findings prompted us to evaluate whether the effects of RHPS4 and 190 on CHK1 expression and RS were accompanied by changes in ERα levels. Both compounds reduced ERα protein levels and *ESR1* mRNA abundance in MCF7 cells in a dose-dependent manner. Together with the protein turnover experiments, these observations suggest that receptor downregulation may involve changes in both gene expression and protein stability. However, they do not establish whether these effects are mediated by CHK1 downregulation or involve additional actions of the ligands. Because ERα signaling supports the proliferation of ERα-positive BC cells [74], we also examined whether this response was retained in an ET-resistant model expressing the ERα Y537S variant, which remains dependent on receptor signaling [75]. Both RHPS4 and 190 reduced *ESR1* mRNA and ERα protein levels in MCF7-Y537S cells, extending the observed response to this endocrine-resistant model.

Our findings also raise the possibility that RHPS4 and 190 affect *ESR1* expression by targeting G4 structures within its promoter. Bioinformatic analysis identified high-scoring putative G4-forming sequence in this region, and CD spectroscopy showed that the corresponding oligonucleotide folds into a stable G4 structure that can be stabilized by both ligands *in vitro*. Together with the decrease in *ESR1* mRNA observed in treated cells, these findings suggest that promoter G4 stabilization may contribute to *ESR1* downregulation. This possible effect on *ESR1* expression could coexist with the proposed contribution of *CHEK1* downregulation and RS to ERα destabilization. Our data, therefore, suggest that the reduction in ERα levels may involve both regulation of gene expression and changes in protein stability, although they do not establish the relative contribution or independence of these processes.

Importantly, the RHPS4 analogues displayed differential abilities to stabilize *CHEK1* and *ESR1* promoter G4s. Among them, compound 190 showed the most favorable profile, exhibiting efficient stabilization of both *CHEK1* and *ESR1* G4 structures while having minimal impact on the telomeric 22AG G4, the canonical target of RHPS4. This reduced affinity for telomeric sequences, together with the preserved activity on promoter G4s, highlights a shift toward enhanced sequence and topology selectivity, and are fully consistent with the cellular data presented in this study. Overall, our data position compound 190 as a promising molecule for the development of next-generation G4 ligands with improved specificity for regulatory promoter G-quadruplexes. Such compounds may enable more targeted modulation of oncogenic transcriptional programs, offering new therapeutic opportunities for luminal and endocrine-resistant breast cancers.

## Material and methods

### Cell lines and culture conditions

BC cell lines MCF7, MCF7-Y537S (CRISPR/CAS9 engineered MCF7 cells expressing the ERα Y537S variant [52]) and MDA-MB-231 triple-negative (ERα^−^, PR^−^, HER2-NEU^−^) were cultured in Dulbecco’s Modified Eagle’s Medium High Glucose (4500 mg/L) supplemented with 10% FBS, 2 mM L-glutamine, 100 U/mL penicillin and 100 µg/mL streptomycin. HCC1937 triple-negative ERα^−^, PR^−^, HER2-NEU^−^ and BRCA1‑mutated cells were grown in RPMI supplemented with 10% FBS. The non-tumorigenic mammary epithelial cell line MCF10A was maintained in DMEM/F12 supplemented with 2 mM L-glutamine, 1% non-essential amino acids, 1 mM sodium pyruvate, 0.5 µg/mL hydrocortisone, 20 ng/mL EGF (Stem Cell technologies, Vancouver, BC, Canada), 100 ng/mL cholera toxin, 100 U/mL penicillin and 100 µg/mL streptomycin. Lung normal human primary fibroblasts MRC5 were maintained in Dulbecco’s modified MEM supplemented with 10% FBS, 2 mM L-glutammine, 100 U/mL penicillin and 100 µg/mL streptomycin. All the cell lines were grown at 37°C in a 5% CO_2_ atmosphere and were routinely tested for mycoplasma contamination using direct staining of mycoplasma’s DNA by DAPI dye. Unless otherwise specified, media, supplements, and plasticware for cell culture were purchased from Euroclone (Euroclone, Pero, MI, Italy).

### Chemical compounds and treatments

Unless otherwise stated, reagents, solvents, and chemicals were purchased from TCI Chemicals and Sigma–Aldrich and used without additional purification.

All the HPLC analyses were carried out using an Agilent system SERIES 1260, using a XSelectHSS C18 (2.5 μm) (50 × 4.6 mm) (Waters) column. Compound identity and purity were assessed by analytical HPLC, ^1^H-NMR spectra recorded on a Bruker ADVANCE 400 MHz and mass spectrometry measurements performed on a Thermo LTQ-XL (linear ion trap mass detector) with an electrospray ionization (ESI) source.

The pentacyclic acridine, 3,11-difluoro-6,8,13-trimethyl-8Hquino [4,3,2-kl] acridinium methosulfate RHPS4 was purchased from Tocris (Bristol, UK; cat. no. 5311). RHPS4-derivative 190 was synthesized in according to a recently optimized published protocol [18], whereas compounds 761 and 785 were prepared by Pharminox Ltd. (Biocity, Pennyfoot St, Nottingham NG1 1GF, UK) and kindly provided to us by Dr. Anna Maria Biroccio [International Patent Application No. PCT/GB2011/051845 and PCT/GB2012/051467]. RHPS4 and its derivatives (190, 761, and 785) were dissolved in dimethyl sulfoxide (DMSO).

If not differently stated, all the experiments were performed treating cells with their respective IC_50_ (concentrations of compound leading to 50% inhibition of cell proliferation) determined 120 h after treatment.

### Sulforhodamine B (SRB) assay

Exponential growing cells seeded in 96-well plates at different cellular density for each cell line (MCF7 cells: 1200 cells/well; Y537S cells: 1000 cell/wells; HCC1937 cells: 6000 cells/well; MDA-MB-231 cells: 1000 cells/well; MCF10A cells: 4000 cell/well; MRC5 cells: ranging from 1000 to 4000 cells/well). The seeding density for each cell line was optimized to ensure exponential growth over the 120 h assay period. Cell proliferation was quantified by SRB staining using an assay adapted from a previously published protocol [15]. For the present study, cells were plated at densities optimized for each cell line and cultured for 120 h before fixation and staining. Cells were fixed in 10% cold trichloroacetic acid (TCA), incubated for 1 h at 4°C and then washed with deionized water. Subsequently, cells were stained for 30 min with 200 µL/well of 0.1% sulforhodamine B (Sigma–Aldrich, St. Louis, MO, USA; cat. no. S1402) and washed with 1% acetic acid. Plates were air-dried at room temperature (RT), and stained proteins were solubilized with 200 µL/well of 10 mM unbuffered Tris base (tris(hydroxymethyl) aminomethane) solution (Sigma–Aldrich, Co., St. Louis, MO, USA). Optical density at 530 nm was read using a Victor plate-reader (VICTOR X3 Multilabel plate reader, PerkinElmer, Waltham, MA, USA). Experiments were repeated at least three times. Data are presented as the mean ± SD.

### Metaphase spreads preparation

Metaphase chromosome preparations were generated using standard cytogenetic procedures. Briefly, cells were exposed to colchicine (5 × 10^−6^ M) for 4 h before collection. After harvesting, cells were incubated in hypotonic 75 mM KCl for 20 min at 37°C and then fixed in freshly prepared Carnoy’s solution (methanol/acetic acid, 3:1 v/v). Fixed cell suspensions were dropped onto glass slides, air-dried, and used for cytogenetic analyses.

### Centromere-calibrated telomeric Q-FISH

Centromere‑calibrated Q-FISH staining was carried out as previously reported [29], with conditions adapted to the present study. Forty-eight hours after the seeding, slides were washed with PBS pH 7.5 and fixed in 4% formaldehyde for 2 min. After two additional PBS washes, slides were dehydrated through graded alcohols. A Cy3-labelled telomeric PNA probe with the sequence (CCCTAA)_3_ (PANAGENE, Daejeon, South Korea) was applied together with a Cy3-labelled PNA probe targeting the chromosome 2 centromere (PANAGENE). Slides and probes were co-denatured at 80°C for 3 min and hybridized for 2 h at RT in a humidified chamber. Following hybridization, slides were washed twice for 15 min in 70% formamide, 10 mM Tris-HCl at pH 7.2, and 0.1% BSA, followed by three 5-min washes in TBS containing 0.08% Tween20. Slides were then dehydrated with an ethanol series and air-dried and counterstained with DAPI in Vectashield mounting medium (Vector Laboratories, Burlingame, CA, USA; cat. no. H-1200). Images were acquired at 63× magnification with an Axio Imager Z2 (Carl Zeiss, Jena, Germany) equipped with a CoolCube 4 Digital High-Resolution CCD camera. The telomere size was calculated using the ISIS software (MetaSystems, Milan, Italy) as the ratio between the fluorescence of each telomere signal and the fluorescence of the centromere of chromosome 2 used as internal reference in each metaphase analyzed. Data were expressed as a percentage (T/C %) ± SD [29,76]. For each sample, at least 30 metaphases have been analyzed in two independent experiments.

### TRF1 and γH2AX co-immunostaining

Cells were fixed with 4% paraformaldehyde, permeabilized with 0.5% TritonX-100 and blocked in PBS containing 1% BSA. Samples were then incubated overnight at 4°C with a rabbit anti-TRF1 antibody (Santa Cruz Biotechnology, Dallas, TX, USA) together with a mouse anti‑phospho-serine 139 γH2AX antibody (Millipore, MA, USA). After washing in PBS/1%BSA, cells were incubated with the secondary antibodies (anti-mouse Alexa 546 and anti-rabbit Alexa 488, Invitrogen). Nuclei were counterstained with DAPI. Fluorescence signals were examined using an Axio-Imager M1 microscope equipped with a digital camera. The number of foci and co-localization events per cell was scored in 100 nuclei for each condition in at least three independent experiments. Data are presented as the mean ± SD.

### Western blot

Whole-cell lysates were prepared in buffer containing 20 mM Tris HCl (pH 7.5), 150 mM NaCl, 1 mM EDTA, 1% Triton-X100, and protease inhibitors. Protein extracts (15 μg) were separated by SDS-PAGE and transferred onto PVDF membranes (0.45 μm pore size; Immobilon-P, Millipore). Membranes were blocked for 30 min at room temperature in TBS containing 3% BSA and 0.05% Tween-20, and then incubated overnight at 4°C with the following primary antibodies: CHK1 (cat. no. sc-8408), ERα (clone F-10; cat. no. sc-8002), and PCNA (clone PC10; cat. no. sc-56), all from Santa Cruz Biotechnology (Dallas, TX, USA); vinculin (cat. no. V9131) and tubulin (cat. no. T0198), both from Sigma–Aldrich (St. Louis, MO, USA); and phospho-RPA2 (Thr21; cat. no. ab109394), phospho-RPA2 (Ser4/Ser8; cat. no. ab243866), phospho-RPA2 (Ser33; cat. no. ab211877), and total RPA2 (cat. no. ab252861), all from Abcam. Finally, membranes were incubated 1 h at room temperature with the appropriate HRP-conjugated secondary antibody (Bio-Rad Laboratories, Hercules, CA, USA). Protein bands were detected by the ChemiDoc enhanced chemiluminescence imaging system (Bio-Rad) and quantified with Image Lab software (Bio-Rad). Band intensities were normalized to the corresponding loading control for non-phosphorylated proteins and to the corresponding total protein levels for phosphorylated proteins. Camptothecin (CPT; Sigma–Aldrich, St. Louis, MO, USA; cat. no. C9911) was used at 100 nM for 24 h. Prexasertib (Selleck Chemicals, USA; cat. no. S6385) was used at 0.01, 0.1, and 1 μM for 24 h. Experiments were performed independently at least three times. Data are presented as the mean ± SD.

### RT-qPCR

Total RNA was isolated using EuroGold Trifast (Euroclone; cat. no. EMR507100) according to the manufacturer’s instructions. Reverse transcription was carried out using oligo-dT primers and the SuperScript™ II Reverse Transcriptase system (Invitrogen, Carlsbad, CA, USA; cat. no. 18,064,014). RNA samples were first incubated at 65°C for 5 min and rapidly chilled on ice, followed by reverse transcription at 50°C for 50 min and enzyme inactivation at 70°C for 15 min. RT-qPCR was performed using SYBR Green PCR Master Mix (Bio-Rad) on an Agilent AriaMx real-time PCR system (Agilent Technologies, Santa Clara, CA, USA). Target gene expression was normalized to GAPDH and calculated using the 2^−ΔΔCt^ method. The following primers were used: *CHEK1* Fw: 5’-CGGTGGAGTCATGGCAGTGCCC-3’; *CHEK1* Rev: 5’-TCTGGACAGTCTACGGCACGCTTCA-3’; *ESR1* Fw: 5’-GTGCCTGGCTAGAGATCCTG-3’; *ESR1* Rev: 5’-AGAGACTTCAGGGTGCTGGA-3’; GAPDH Fw: 5′-AGCCACATCGCTCAGACAC-3′; GAPDH Rev: and 5′-GCCCAATACGACCAAATCC-3′.

Data are reported as fold change relative to untreated controls. Experiments were performed in triplicate. Data are presented as the mean ± SD.

### Bioinformatic studies

Putative G-quadruplex-forming sequences (PQS) were identified using G4Hunter [77] and QGRS Mapper, computational algorithms that predict G4 propensity based on local guanine content and sequence asymmetry. The genomic regions encompassing the *CHEK1* and *ESR1* promoters were retrieved from the *Homo sapiens* reference genome (GRCh38.p14) and analyzed using a sliding window of 25 nucleotides and a threshold value of 2.0. For *CHEK1*, the nucleotide region *Chr11: 125,624,800-125681124* was analyzed. The *CHEK1* promoter core region (*Chr11:125624800– 125,628,201*), corresponding to positions 1–3402 in the analyzed sequence, was specifically examined, and the PQS displaying the highest G4 Hunter score (2.038), corresponding to the sequence 5′-G_3_ACG_3_TG_4_CTGTG_2_ATG_3_A-3′, was selected for further investigation. For *ESR1*, the nucleotide region *Chr6:151656672– 152,129,619* was scanned under the same parameters. The PQS displaying the highest G4Hunter score (2.115), corresponding to the sequence 5′-G_3_AG_3_AG_3_AG_3_AG_3_AGA_2_G_3_-3′, was identified and selected for subsequent experimental characterization.

### Sample preparation

*ESR1* (5’-GGGAGGGAGGGAGGGAGGGAGAAGGG-3’) and *CHEK1* (5’-GGGACGGGTGGGGCTGTGGATGGGA-3’) model oligonucleotides were purchased from Sial s.r.l. (Sial srl, Rome, Italy) with HPLC-grade purity and used without further purification. They were dissolved in MilliQ water to 500 µM stock solutions and stored at −20°C. For working solutions, stocks were diluted to a final concentration of 2.5 µM in 10 mM lithium cacodylate buffer (pH 7.4) containing different KCl concentrations (10, 20, and 100 mM). Ionic strength was maintained constant at 100 mM by adding proper amounts of LiCl. Samples were thermally annealed by heating at 95°C for 5 min, followed by slow cooling to RT over 4 h to allow G4 folding.

### Circular dichroism (CD) measurements

CD spectra were collected on a JASCO J-1500 spectropolarimeter at 310 K using a 1 cm path-length quartz cuvette. Data were acquired in the 210–340 nm range with a 1 nm step size, 2 s response time, and a scanning speed of 200 nm min^−1^. Each spectrum represents the average of four accumulations and was baseline-corrected for buffer contributions. Ellipticity values (θ, in millidegrees) were converted to molar ellipticity (Δε, M^−1^ cm^−1^) according to the equation Δε = θ / (32,980 × C × l), where C is the oligonucleotide molar concentration and l is the optical path length in centimeters.

For CD-melting experiments, the ellipticity of 2.5 µM G4 solutions was monitored at 264 nm over a temperature range of 20–95°C, using a heating rate of 1°C min^−1^ and data acquisition every 0.5°C.

Specifically, to guarantee a melting temperature of the oligonucleotides alone equal to around 60°C, 10 mM KCl was used for *ESR1*, as well as 20 mM KCl for *CHEK1*. Ligands (RHPS4, 190, 761, and 785) were added to a final concentration of 10 µM (4 equiv.) and incubated for 4 h at RT before thermal analysis. Melting temperatures (Tm) were determined using the Van’t Hoff approximation for a two-state transition (folded-unfolded), assuming identical heat capacities for both states.

### Chromatin immunoprecipitation

Chromatin immunoprecipitation (ChIP) was performed on cells treated for 24 h with RHPS4 and 190 and grown to confluence in 145-mm culture dishes. Cells were cross-linked directly in the culture medium with 1% formaldehyde for 10 min at room temperature and the reaction was quenched with 0.125 M glycine for 5 min. Following washing with ice-cold PBS, cells were collected by scraping and pelleted by centrifugation. Cell pellets were lysed in Cell Lysis Buffer (5 mM PIPES, pH 8.0, 85 mM KCl, 0.5% NP-40) supplemented with protease inhibitors, and nuclei were subsequently lysed in SDS Lysis Buffer (50 mM Tris-HCl, pH 8.0, 10 mM EDTA, 1% SDS). Chromatin was sheared by sonication using a Bioruptor sonicator (Diagenode) to generate DNA fragments ranging from 400 to 800 bp. Sonication efficiency was assessed after reverse cross-linking of an aliquot of chromatin and analysis by agarose gel electrophoresis. After removal of insoluble debris, chromatin concentration was determined, and 100 μg of chromatin was diluted in ChIP Dilution Buffer (16.7 mM Tris-HCl, pH 8.0, 167 mM NaCl, 0.01% SDS, 1.1% Triton X-100, 1.2 mM EDTA). Samples were pre-cleared with magnetic beads for 1 h at 4°C and incubated overnight at 4°C with 4 μg of mouse anti‑DNA/RNA G-quadruplex antibody (clone BG4; Absolute Antibody; cat. no. Ab00174-1.1). Parallel immunoprecipitations performed with 4 μg of normal mouse IgG (MA5-54746, Thermo Fisher Scientific, Waltham, MA, USA) served as negative controls. Immune complexes were captured using 60 μl magnetic beads (Dynabeads Protein G 10003D, Thermo Fisher Scientific) and washed sequentially with Low Salt Wash Buffer (20 mM Tris-HCl, pH 8.0, 2 mM EDTA, 150 mM NaCl, 1% Triton X-100, 0.1% SDS), High Salt Wash Buffer (20 mM Tris-HCl, pH 8.0, 2 mM EDTA, 500 mM NaCl, 1% Triton X-100, 0.1% SDS), LiCl Wash Buffer (10 mM Tris-HCl, pH 8.0, 1 mM EDTA, 0.25 M LiCl, 1% NP-40, 1% sodium deoxycholate), and TE buffer. Chromatin was eluted in Elution Buffer (1% SDS, 0.1 M NaHCO_3_), and cross-links were reversed by incubation with NaCl at 65°C overnight. Samples were treated with 2 μl RNase A (10 mg/ml EN0531, Thermo Fisher Scientific) and Proteinase K (10 mg/ml 4,333,793, Thermo Fisher Scientific), and DNA was purified by phenol:chloroform:isoamyl alcohol extraction followed by ethanol precipitation. Purified DNA was resuspended in nuclease-free water and analyzed by quantitative real-time qPCR with the following primers: *MDM2* Fw: GGA TTT CGG ACG GCT CTC G; *MDM2* Rev: CGT TCA CAC TAG TGA CCC GA; *CHEK1* Fw: CCA GCA GCG CTC GAG CAC C; *CHEK1* Rev: AGA CAG CGC GCG TTC CCG TG. Data are presented as the mean ± SD.

### Statistical analysis

Statistical analyses were performed using GraphPad Prism 10.5.0. Student’s t-test was used for comparisons between two groups, whereas one-way or two-way analysis of variance (ANOVA), as appropriate, followed by Tukey’s multiple-comparison test was used for comparisons among multiple groups. The number of independent biological replicates, measures of variability, and statistical tests used are specified in the corresponding figure legends. *p* values < 0.05 were considered statistically significant and are indicated as follows: *p < 0.05, **p < 0.01, ***p < 0.001, and ****p < 0.0001.

## Supplementary Material

Supplementary materials and methods.docx

Supplementary Tables.docx

Supplementary information.docx

## Data Availability

The data supporting the findings of this study are available from the corresponding author upon reasonable request.

## Abbreviations

ET: Endocrine Therapy
PARPi: PARP inhibitors
ERα: Estrogen Receptor α
SERMs: Selective ERα modulators
SERDs: Selective ERα downmodulators
TNBC: Triple Negative Breast Cancer
BC: Breast cancer
Q-FISH: Quantitative Fluorescence In Situ Hybridization
WB: Western blotting
RFS: Relapse Free Survival
CPT: Camptothecin
CHX: Cycloheximide
CD: Circular Dichroism
EGF: Epidermal Growth Factor
ESI: Electrospray Ionization
DMSO: Dimethyl Sulfoxide
SRB: Sulforhodamine B
PVDF: Polyvinylidene Fluoride
PQS: Putative G-Quadruplex-forming Sequences
