# Supplementary material for: G-quadruplex ligand-mediated CHK1 and ERα suppression in endocrine-sensitive and endocrine-resistant luminal breast cancer cells
Source: Cell Cycle. 2026 Oct 30;25(1):1–22. doi: 10.1080/15384101.2026.2753856 (PMC13733213; doi:10.1080/15384101.2026.2753856)
Supplement: Supplementary file 1 [file KCCY_A_2753856_SM7122.docx]

**Supplementary Figures**

**Figure S1.** Scatter plots representing relation between RHPS4 or 190 IC_50_ (μM) and mean telomere length (T/C%)(A, E), fraction of short telomeres (B, F), 𝛾H2AX frequency (C, G) and basal TIF frequency (D, H) in normal and BC cells. Strong positive correlations were found for both TIF and 𝛾H2AX (R^2^ = 0.82 and 0.62 for 𝛾H2AX-IC_50_ correlation in cells treated with RHPS4 and 190, respectively; R^2^ = 0.80 and 0.77 for TIF-IC_50_ correlation in cells treated with RHPS4 and 190, respectively).

**Figure S2.** Representative western blot showing CHK1 and PCNA protein amounts in untreated (CTRL) and G4 ligands-treated HCC1937 cells (A). Vinculin was used to ensure for equal protein loading. Quantification of CHK1 (B) protein amounts after 190 and RHPS4 treatment. Data have been reported as relative protein levels with respect to untreated cells and represent mean values ± SD (n = 3). Quantification of *CHEK1* mRNA expression upon RHPS4 or 190 exposures (C). Data have been reported as relative quantity with respect to untreated cells, according to the 2-ΔΔCt method, and represent mean values ± SD (N = 3). **P* < 0.05, ***P* < 0.01, ****P* < 0.001 (One way ANOVA test and Tukey’s multiple comparison test).

**Figure S3.** Representative profiles of MCF-7 (A), MCF-7-Y537S (B) and HCC1937 (C) cells analyzed by cytofluorimetry following a BrdU pulse and chase approach upon treatment. Note that G1 BrdU positive cells appear at 8 hours and continue to accumulate till 24 h in untreated cells, whereas in treated samples, they appear only after 24h (or did not appear at all) indicating a delay in the cell cycle. However, no S-phase depletion was observed upon RHPS4 or 190 treatments in BC cells analyzed. Histograms show the quantitation of BrdU positive and negative G1 cells in MCF7 (D), MCF7-Y537S (E) and HCC1937 (F) treated with RHPS4 and 190 in comparison to untreated cells. (Data have been reported as % cell and represent mean values ± SD, N = 3). **P* < 0.05, ***P* < 0.01, ****P* < 0.001, *****P* <0.0001 (Student's *t*-test).

**Figure S4.** 3D plots showing that RHPS4- and 190-dependent (A,B) reduction of ERα is accompanied by an increase in RPA (at S33, S4_8 and T21) and H2AX (S139) phosphorylation. Scatter plots representing linear correlation between RHPS4- and 190-dependent (C, D) reduction of cell proliferation rate and ERα protein levels (R^2^ = 0.96 and 0.98, for RHPS4 and 190 treatments, respectively). Error bars denote SD.

**Figure S5.** Western blot (A, C) and relative densitometric analysis (B, D) of ERα levels in MCF7 cells pre-treated with cycloheximide (CHX) at the indicated concentrations. The loading control was done by evaluating vinculin (VINC) expression in the same filter. The number of replicates is given as open dots in the bar graph (N=4 for RHPS4; N=5 for 190). Error bars denote standard deviations (SD). Significant differences with respect to CTRL or CHX sample are calculated by Student t-test and indicated by * (p-value < 0.05) *** (p-value < 0.001) and **** (p-value < 0.0001).

**Figure S6.** Principal component analysis of CD spectra from *CHEK1* and *ESR1* sequences compared with reference G4 structures of known topology. PCA was performed using a dataset of experimental CD spectra collected from structurally characterized G4s [1], allowing discrimination among parallel (yellow), hybrid (blue), and antiparallel (green) topologies. CD spectrum of *ESR1* with 10 mM KCl clusters within the region of parallel G4s, whereas the one of *CHEK1* with 20 mM KCl falls within the hybrid G4 cluster.

E

C

D

A

B

**Figure S7.** CD-melting profile of 2.5 μM *CHEK1* (A, B) or *ESR1* (C, D) in 10 mM lithium cacodylate buffer, pH 7.4, at different KCl concentration (10-20 mM and 5-10 mM for *CHEK1* and *ESR1*, respectively), followed at 264 nm within a temperature range of 20-95°C (A-D). CD-melting profile of 2.5 μM 22AG in 10 mM lithium cacodylate buffer, pH 7.4, 20 mM KCl, followed at 292 nm within a temperature range of 20-95°C (E).

B

A

D

C

E

F

H

G

**Figure S8**. Data processing to define thermodynamic parameters from UV-melting experiments of 2.5 µM ESR1 G4 in 10 mM lithium cacodylate buffer pH 7.4, with 5 mM KCl and 95 mM LiCl. A) Folded fraction calculation; B) A Van’t Hoff representation by restricting temperature range for which folded fraction is 0.03 <ø < 0.97. Data processing to define thermodynamic parameters from UV-melting experiments of 2.5 µM ESR1 G4 in 10 mM lithium cacodylate buffer pH 7.4, with 10 mM KCl and 90 mM LiCl. C) Folded fraction calculation; D) A Van’t Hoff representation by restricting temperature range for which folded fraction is 0.03 <ø < 0.97. Data processing to define thermodynamic parameters from UV-melting experiments of 2.5 µM CHEK1 G4 in 10 mM lithium cacodylate buffer pH 7.4, with 10 mM KCl and 90 mM LiCl. E) Folded fraction calculation; F) A Van’t Hoff representation by restricting temperature range for which folded fraction is 0.03 <ø < 0.97. Data processing to define thermodynamic parameters from UV-melting experiments of 2.5 µM CHEK1 G4 in 10 mM lithium cacodylate buffer pH 7.4, with 20 mM KCl and 80 mM LiCl. G) Folded fraction calculation; H) A Van’t Hoff representation by restricting temperature range for which folded fraction is 0.03 <ø < 0.97.

**Figure S9.** CD spectra of 2.5 μM of *CHEK1* (A) or *ESR1* (B) in 10 mM lithium cacodylate buffer, pH 7.4, with 20 mM or 10 mM KCl, respectively, and 4 equivalents of ligands. The ionic strength was maintained constant to 100 mM adding proper amount of LiCl.

**Figure S10.** Surviving Fraction (SF) of MCF7 (A) and MCF7-Y537S (B) cells pretreated with (or without) RHPS4 and 190 (IC_50_ for 120 h) and irradiated with 2.5 and 5 Gy X-rays. No significant difference in radiosensitivity was observed comparing untreated and G4-ligands pretreated MCF-7 and MCF-7-Y537S cells (Data have been reported as fraction of surviving cells and represent mean values ± SD, N = 3).

**Reference list**

1. Del Villar-Guerra R, Trent JO & Chaires JB (2018) G-Quadruplex Secondary Structure Obtained from Circular Dichroism Spectroscopy, Angew Chem Int Ed Engl. 57, 7171-7175.
