# Supplementary material for: G-quadruplex ligand-mediated CHK1 and ERα suppression in endocrine-sensitive and endocrine-resistant luminal breast cancer cells
Source: Cell Cycle. 2026 Oct 30;25(1):1–22. doi: 10.1080/15384101.2026.2753856 (PMC13733213; doi:10.1080/15384101.2026.2753856)
Supplement: Supplementary file 2 [file KCCY_A_2753856_SM7123.docx]

**Table S1.** Tm values were obtained in the presence of 2.5 µM oligonucleotides with 4 equiv. of RHPS4, 190, 761, or 785 (10 µM) in lithium cacodylate buffer (10 mM, pH 7.4) with different concentrations of KCl.

|  |  | **Tm (°C) by CD-melting** | | | |
| --- | --- | --- | --- | --- | --- |
|  | **[KCl] (mM)** | **+RHPS4** | **+190** | **+761** | **+785** |
| ***ESR1*** | 10 | 80.7 ± 0.6 | 76.1 ± 0.6 | > 95 | > 95 |
| ***CHEK1*** | 20 | 79.3 ± 0.5 | 80.4 ± 0.4 | 86.6 ± 0.9 | 87.3 ± 0.6 |
| **22AG** | 20 | 85.7 ± 0.7 | 61.7 ± 0.1 | 73.0 ± 0.2 | 76.6 ± 0.2 |

**Table S2.** ∆Tm values were obtained in the presence of 2.5 µM oligonucleotides with 4 equiv. of RHPS4, 190, 761, or 785 (10 µM) in lithium cacodylate buffer (10 mM, pH 7.4) with different concentrations of KCl.

|  |  | **ΔTm (°C) by CD-melting** | | | |
| --- | --- | --- | --- | --- | --- |
|  | **[KCl] (mM)** | **+RHPS4** | **+190** | **+761** | **+785** |
| ***ESR1*** | 10 | 14.8 ± 0.8 | 15.9 ± 0.8 | > 30.5 | > 30.5 |
| ***CHEK1*** | 20 | 15.0 ± 0.8 | 11.6 ± 0.7 | 22 ± 1 | 22.8 ± 0.9 |
| **22AG** | 20 | 29.6 ± 0.8 | 5.7 ± 0.2 | 17.0 ± 0.3 | 20.6 ± 0.3 |
