# Supplementary material for: G-quadruplex ligand-mediated CHK1 and ERα suppression in endocrine-sensitive and endocrine-resistant luminal breast cancer cells
Source: Cell Cycle. 2026 Oct 30;25(1):1–22. doi: 10.1080/15384101.2026.2753856 (PMC13733213; doi:10.1080/15384101.2026.2753856)
Supplement: Supplementary file 3 [file KCCY_A_2753856_SM7124.docx]

**Supplementary materials and methods**

**Flow Cytometric Analysis**

After treatments with RHPS4 or 190 (using their respective IC_50_) for 120 hours, cells were pulsed 30 min with 10 µM BrdU, then washed and harvested immediately or grown in fresh medium and harvested after 8 and 24 h. Cells were then fixed, permeabilized, and the histones were dissociated with 2 M HCl as previously described [1]. BrdU positive cells were detected with an anti-BrdU primary antibody diluted 1:100 (DAKO Cytomatation, Glostrup, Denmark) and with an anti-mouse-Alexa488 conjugated antibody diluted 1:100 (Invitrogen, Life Technologies, Carlsbad, CA, USA). Both antibodies were incubated for 1 h RT in the dark. All samples were counterstained with propidium iodide (PI) for DNA/BrdU biparametric analysis. Samples were acquired using a CytoFLEX flow cytometer (Beckman Coulter, Brea, CA, USA). BrdU positive cells were gated, and the relative percentage was calculated by Cytexpert software (Beckman Coulter, Inc., San Diego, CA, USA).

**Irradiation conditions *in vitro***

Cells were seeded (10,000 cells/cm²) and grown in T75 flasks or 6 cm Petri dishes (Euroclone, Pero, MI, Italy). After treatment with RHPS4 or 190 for 120 hours, cells were irradiated using an X-ray tube (Gilardoni) at 250 kV and 6 mA. The doses used were 2.5 and 5 Gy, with a dose rate of 0.53 Gy/min.

**Colony-forming assay**

To evaluate clonogenic survival, untreated and treated (with RHPS4 or 190) U251MG, MCF7, MCF7-Y537S and MDA-MB-231 cells were irradiated with 2.5 and 5 Gy of X-rays and then plated at appropriate cell densities in 6-well plates. After 15 days, cells were fixed/stained with an aqueous solution containing 0.25% (w/v) crystal violet, 70% (v/v) methanol and 3% (v/v) formaldehyde, and they were counted. Only colonies comprised of > 50 cells were included in the quantification. “For each treatment, the surviving fraction (SF) was calculated as follows: SF = number of colonies formed/(number of cells seeded × plating efficiency of the corresponding non-irradiated control). Plating efficiency was calculated as the ratio of colonies formed to cells seeded in the corresponding non-irradiated condition. Results were reported as the mean of three independent experiments.”

**UV-Visible Absorption Studies**

UV-Vis measurements were performed on an Agilent Cary 60 spectrophotometer equipped with a single-cell Peltier temperature controller, using a 1 cm quartz cuvette. Oligonucleotides were diluted to a final concentration of 2.5 µM in 10 mM lithium cacodylate buffer (pH 7.4) containing different KCl concentrations (5, 10, and 20 mM). Ionic strength was maintained constant at 100 mM by adding proper amounts of LiCl. Absorbance spectra were recorded between 220 and 340 nm, with a scan rate of 50 nm min⁻¹ and a slit width of 0.5 nm, while the temperature was increased from 20 to 95 °C.

1. Amato R, Micheli E, Focaccetti C, Bei R, La Rosa P, Sgura A, Berardinelli F. (2026) Telomeric Replication Stress Promotes ALT-Associated Hallmarks in ATRX- and p53-Deficient Cells. Front. Biosci. Landmark **31**, 52954.
